# Enhancing Dysarthric Voice Conversion with Fuzzy Expectation Maximization in Diffusion Models for Phoneme Prediction

**DOI:** 10.3390/diagnostics14232693

**Published:** 2024-11-29

**Authors:** Wen-Shin Hsu, Guang-Tao Lin, Wei-Hsun Wang

**Affiliations:** 1 Department of Medical Information, Chung Shan Medical University, Taichung 402201, Taiwan; wshsu@csmu.edu.tw (W.-S.H.); todlin89@gmail.com (G.-T.L.); 2 Informatics Office Technology, Chung Shan Medical University Hospital, Taichung 402201, Taiwan; 3 College of Medicine, National Chung Hsing University, Taichung 402202, Taiwan; 4 Department of Golden-Ager Industry Management, Chaoyang University of Technology, Taichung 413310, Taiwan; 5 Department of Orthopedic Surgery, Changhua Christian Hospital, Changhua 500209, Taiwan; 6 Department of Medical Imaging and Radiology, Shu-Zen Junior College of Medicine and Management, Kaohsiung 82100, Taiwan

**Keywords:** dysarthria, voice conversion, Fuzzy Expectation Maximization, diffusion models, assistive communication technology

## Abstract

**Introduction:** Dysarthria, a motor speech disorder caused by neurological damage, significantly hampers speech intelligibility, creating communication barriers for affected individuals. Voice conversion (VC) systems have been developed to address this, yet accurately predicting phonemes in dysarthric speech remains a challenge due to its variability. This study proposes a novel approach that integrates Fuzzy Expectation Maximization (FEM) with diffusion models for enhanced phoneme prediction, aiming to improve the quality of dysarthric voice conversion. **Methods:** The proposed method combines FEM clustering with Diffusion Probabilistic Models (DPM). Diffusion models simulate noise addition and removal to enhance the robustness of speech signals, while FEM iteratively optimizes phoneme boundaries, reducing uncertainty. The system was trained using the Saarland University Voice Disorder dataset, consisting of dysarthric and normal speech samples, with the conversion process represented in the Mel-spectrogram domain. The framework employs both subjective (Mean Opinion Score, MOS) and objective (Word Error Rate, WER) metrics for evaluation, complemented by ablation studies. **Results:** Experimental results showed that the proposed method significantly improved phoneme prediction accuracy and overall voice conversion quality. It achieved higher MOSs for naturalness, intelligibility, and speaker similarity compared to existing models like StarGAN-VC and CycleGAN-VC. Additionally, the proposed method demonstrated a lower WER for both mild and severe dysarthria cases, indicating better performance in producing intelligible speech. **Discussion:** The integration of FEM with diffusion models offers substantial improvements in handling the irregularities of dysarthric speech. The method’s robustness, as evidenced by the ablation studies, shows that it can maintain speech naturalness and intelligibility even without a speaker-encoder. These findings suggest that the proposed approach can contribute to the development of more reliable assistive communication technologies for individuals with dysarthria, providing a promising foundation for future advancements in personalized speech therapy.

## 1. Introduction

Dysarthria [1,2,3,4] is a motor speech disorder characterized by disruptions in the coordination and execution of the speech production process. It can be categorized based on the underlying neuropathology affecting different components of speech production, including respiration, laryngeal function, airflow direction, and articulation. This disorder leads to significant challenges in speech quality and clarity.

1. There are six primary types of dysarthria, each associated with different neurological impairments: [5,6,7]
2. Flaccid Dysarthria: Results from lower motor neuron impairment, which leads to weakness and reduced muscle tone, affecting speech production. Individuals may exhibit breathy or weak voice quality, imprecise articulation, and reduced intelligibility [8,9].
3. Spastic Dysarthria: Associated with damage to upper motor neurons in the motor areas of the cerebral cortex. This type typically causes strained, strangled speech quality, imprecise articulation, and a slow rate of speech due to increased muscle tone and spasticity [8].
4. Ataxic Dysarthria: Primarily caused by cerebellar dysfunction, leading to a lack of coordination and control over speech movements. This type is characterized by irregular articulatory breakdowns, variable speech rate, and a jerky, uneven quality of speech [10].
5. Hyperkinetic Dysarthria: Linked to disorders of the extrapyramidal system, such as Huntington’s disease, this type features involuntary movements that disrupt speech production, leading to irregular pitch, loudness variations, and erratic speech rhythm [11].
6. Hypokinetic Dysarthria: Also related to extrapyramidal system disorders, particularly Parkinson’s disease, this type is marked by reduced range of motion, slow speech rate, and a monotonous voice due to diminished movement amplitude [12].
7. Mixed Dysarthria: Involves a combination of symptoms from two or more types of dysarthria, typically resulting from damage across multiple neurological areas. Speech characteristics will reflect a mix of different dysarthria types, complicating diagnosis and treatment [1].

Diagnosis of dysarthria involves a comprehensive evaluation, including speech assessments, neurological examinations, and possibly imaging studies, to determine the type and extent of the disorder. Management and treatment strategies for dysarthria aim to improve communication abilities and may include speech therapy, medical interventions, and the use of assistive technologies. Speech therapy focuses on exercises and techniques to enhance speech clarity, control, and strength. The impact of dysarthria extends beyond speech clarity; it profoundly affects an individual’s ability to communicate effectively, which is crucial for expressing one’s personality and emotions, and maintaining social connections. For many patients and their families, dysarthria poses significant challenges, making effective management and support essential for improving quality of life.

Patients with dysarthria sometimes require surgical interventions to address structural abnormalities in the speech mechanism, such as vocal fold repairs or surgical modifications to the larynx. However, it is important to note that surgical outcomes do not always result in significant improvements in speech intelligibility or overall dysarthria severity. The complexity of dysarthria, with its multifaceted neurological origins and varied manifestations, means that surgery alone may not always address the underlying issues or improve communication effectively. Recent advancements in technology have provided promising solutions for managing dysarthria through innovative applications. One notable development is the MMSE DiscoGAN [13], a specialized deep learning model designed to enhance the intelligibility of dysarthric speech. This model leverages generative adversarial networks (GANs) to produce more natural-sounding speech, offering an improvement over the traditional deep neural network-based model. MMSE DiscoGAN’s ability to generate clearer and more natural speech can significantly aid individuals with dysarthria in communicating more effectively. Additionally, there have been significant strides in the development of methods for detecting and classifying the severity of dysarthria. Researchers have proposed various approaches to improve the accuracy and reliability of dysarthria assessments. For example, M. Suresh [14] et al. and Joshy et al. [15] have demonstrated that deep neural network (DNN) models can achieve higher accuracy in classifying the severity of dysarthria compared to traditional classifiers. These advanced models utilize sophisticated algorithms to analyze speech patterns and identify subtle variations in speech characteristics, leading to more precise and nuanced assessments of dysarthria severity. 

Recent advancements in voice conversion technology have introduced Duta-VC [16], a state-of-the-art Dysarthria Voice Conversion (DVC) system built upon the Diffusion Probabilistic Model. This innovative system harnesses the power of forward and reverse diffusion techniques, enabling it to achieve significant improvements in speech intelligibility for individuals with dysarthria. One of the most notable features of Duta-VC is its ability to enhance speech clarity without the need for parallel data training, a common requirement in traditional voice conversion models. Additionally, Duta-VC excels in preserving the speaker’s identity, ensuring that the converted speech remains recognizable and true to the original speaker. This advanced approach not only improves the overall quality of dysarthric voice conversion but also addresses specific speech features such as phonemes, formant valleys, resonance bands, and more. These improvements are achieved under a mechanism of duration awareness, which ensures that the temporal aspects of speech—such as the timing and length of phonemes—are accurately maintained, resulting in more natural and intelligible speech output.

In the realm of pattern recognition, K-means clustering remains a widely utilized method due to its simplicity and efficiency. Introduced by MacQueen [17], the original K-means algorithm is particularly favored for its straightforward approach to data clustering. However, despite its popularity, K-means has certain limitations. The convergence of the algorithm to a local minimum, as demonstrated in the variant proposed by Forgy, depends heavily on the initial seed selection [18,19]. While increasing the number of seeds can enhance the chances of reaching an optimal solution, there is no guarantee of achieving the best possible clustering outcome. This inherent uncertainty highlights the need for improvements to the basic K-means algorithm, especially when dealing with complex or noisy data. One of the primary challenges with K-means is its assumption that clusters are spherical and noise-free, which may not hold true in many real-world scenarios. Moreover, the algorithm’s performance is highly sensitive to the initial configuration, requiring precise initialization to function effectively. To overcome these limitations, various enhancements to K-means have been proposed, aimed at achieving more stable and meaningful clusters. S. Nasser et al. [20] discuss these limitations and suggest methods to refine K-means clustering for better stability and accuracy. By integrating the Expectation Maximization (EM) algorithm with K-means, a more sophisticated clustering method can be developed [21,22]. This combined approach not only determines the number of clusters more effectively but also ensures that the clusters are compact and statistically significant. The fusion of statistical techniques with fuzzy logic theory has further demonstrated promising results in improving clustering outcomes.

While K-means is popular, other clustering algorithms, such as Hierarchical Clustering, Gaussian Mixture Models (GMM), and Fuzzy C-means [23] have been applied in various fields to address limitations of K-means, especially when dealing with non-spherical clusters or overlapping data. Hierarchical Clustering does not require a predefined number of clusters and is effective for capturing nested structures in data. GMM assumes that data points are generated from a mixture of several Gaussian distributions, offering flexibility in capturing more complex shapes of clusters. Fuzzy C-means extends K-means by allowing data points to belong to multiple clusters with varying degrees of membership, which is particularly useful in applications like speech processing where boundaries between clusters can be ambiguous.

Building on these advancements, the proposed fuzzy expectation-maximization phoneme prediction method in the diffusion model-based dysarthria voice conversion (FEMPPDM-DVC) method represents a novel integration of the FEM clustering algorithm with Duta-VC. This approach aims to provide a more robust and accurate conversion of articulation abnormalities, particularly those arising from different etiologies. By leveraging the strengths of FEM clustering, the FEMPPDM-DVC method enhances the ability to capture and address the diverse speech patterns observed in dysarthria, leading to more effective and personalized voice conversion solutions.

## 2. Materials and Methods

**Figure 1:**
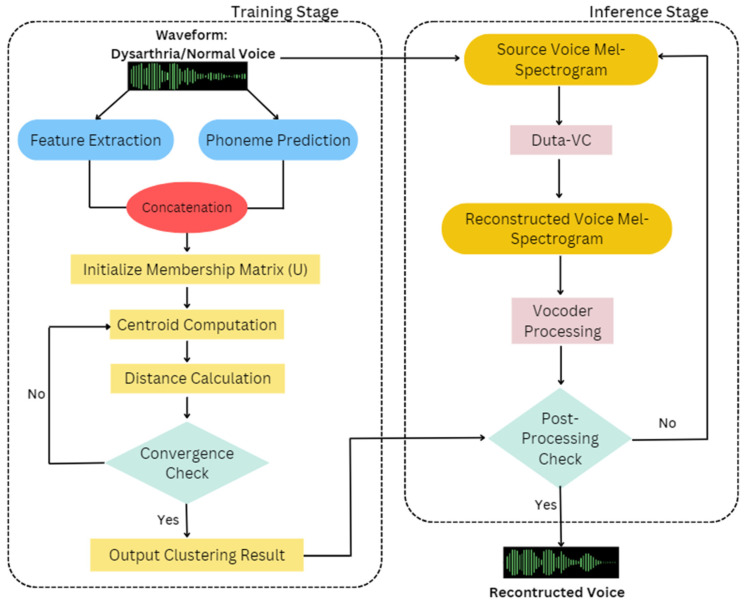
FEMPPDM-DVC Structural Flowchart.

In this section, we delve into the specifics of the datasets utilized and the proposed FEMPPDM-DVC method. The core concept underlying this approach is the conversion of the original voice into a target dysarthric voice within the time-frequency domain, using Mel-spectrograms as the primary representation rather than raw waveforms. Mel-spectrograms provide a more compact and informative representation of speech, capturing essential features such as pitch, intensity, and timbre, which are crucial for accurately modeling and converting dysarthric speech. The FEMPPDM-DVC method is built upon a conditional Diffusion Probabilistic Model (DPM) [24,25,26,27,28,29,30,31,32,33,34,35,36,37,38] that incorporates a data-dependent prior. This model operates through two key processes: forward diffusion and reverse diffusion. In the forward diffusion process, Gaussian noise is incrementally added to the input data, progressively degrading the original speech signal. This process effectively transforms the clean Mel-spectrogram into a noisy version, which the model then learns to reverse. The reverse diffusion process is tasked with denoising the corrupted Mel-spectrogram, aiming to reconstruct a clear and intelligible version of the target dysarthric speech. The training of the model focuses on minimizing the discrepancy between the trajectories of the forward and reverse diffusion processes. By reducing this gap, the model becomes adept at converting the original voice into a dysarthric voice with high fidelity, maintaining both the intelligibility and the speaker’s unique vocal characteristics. The structural framework of the FEMPPDM-DVC method is depicted in Figure 1. This diagram illustrates the intricate processes involved in the voice conversion pipeline, highlighting the roles of both the FEM clustering algorithm and the DPM in achieving precise and personalized voice conversion [24,27,39,40,41,42].

The flowchart is divided into two main stages: the Training Stage (top section) and the Inference Stage (bottom section), visually distinct by background shading.

1. Training Stage
  - Waveform Input: The process begins with the input of an audio waveform, representing either dysarthric or normal speech.
  - Feature extraction: Key features are extracted from the waveform, which includes essential elements like Mel-Frequency Cepstral Coefficients (MFCCs). To enhance temporal dynamics, the feature vector integrates both the first (delta) and second (delta-delta) derivatives of MFCCs. As a result, the complete feature vector comprises multi-dimensions, encompassing the original MFCCs along with their delta and delta-delta coefficients. The MFCC’s feature is as follows:
    $$X_{MFCC}^{\left(t\right)}=\left[x_{MFCC,\;1}^{\left(t\right)},x_{MFCC,\;2}^{\left(t\right)},\ldots ,x_{MFCC,\;d_{MFCC}}^{\left(t\right)}\right]$$
    where t∈{1, 2,…,T}, for the MFCC features of frame t, we denote them as vector $X_{MFCC}(t)$, which is a $d_{MFCC}$-dimensional vector.
  - Phoneme Prediction: The FastSpeech-based phoneme predictor uses a feed-forward transformer (FFT) architecture, combining self-attention in the transformer and 1D convolution. The FFT structure has N blocks each for phoneme-to-Mel-spectrogram transformation, separated by a length regulator to address sequence length differences. Each FFT block integrates self-attention with multi-head attention for cross-position information, and a 2-layer 1D convolutional network with ReLU activation, replacing the dense network in the original transformer [43]. The feature is jointly optimized with a phoneme predictor using a frame-level cross-entropy (CE) loss during training. The ground truth phoneme labels and durations are obtained through forced alignment. The extracted features are then used to predict phonemes, contributing to the analysis of speech patterns. The phoneme prediction feature is as follows:
    $$X_{phoneme}^{\left(t\right)}=\left[x_{phoneme,1}^{\left(t\right)},x_{phoneme,\;2}^{\left(t\right)},\ldots ,\;x_{phoneme,\;d_{phoneme}}^{\left(t\right)}\right]$$
    for the phoneme label of frame t, we represent it as a vector $X_{phoneme}(t)$, which is a $d_{phoneme}$-dimensional vector.
  - Concatenation: The outputs from feature extraction and phoneme prediction are concatenated to form a unified dataset, which serves as the input for the clustering algorithm.
    $$X^{\left(t\right)}=\left[X_{MFCC}^{\left(t\right)},X_{phoneme}^{\left(t\right)}\right]$$
  - After expansion, we can express it as:
    $$X^{\left(t\right)}=\left[x_{MFCC,\;1}^{\left(t\right)},x_{MFCC,\;2}^{\left(t\right)},\ldots ,x_{MFCC,d_{MFCC}}^{\left(t\right)},x_{phoneme,\;1}^{\left(t\right)},x_{phoneme,\;2}^{\left(t\right)},\ldots ,x_{phoneme,\;d_{phoneme}}^{\left(t\right)}\right]$$
  - Initialize Membership Matrix (U): The FEM clustering process starts with the random initialization of the membership matrix, denoted as *U*.
  - Centroid Computation: The centroids for each cluster are computed based on the initialized membership matrix, providing a reference point for each cluster in the data.
  - Distance Calculation: The distances between each data point and the computed centroids are calculated, guiding the reassignment of membership degrees.
  - Convergence Check: The process iteratively checks for convergence by examining whether the differences in the membership matrix fall below a predefined threshold (e.g., Frobenius norm).
  - If not converged: The process repeats the steps of centroid computation and distance calculation.
  - If converged: The final clustering results are output based on the stable membership matrix.
2. Inference Stage
  - Source Voice Mel-Spectrogram: The source voice is converted into a Mel-spectrogram, which serves as the input for the Duta-VC model.
  - Duta-VC: The Duta-VC model processes the source Mel-spectrogram, transforming it into a reconstructed voice Mel-spectrogram that aligns with the target voice characteristics.
  - Vocoder Processing: The reconstructed Mel-spectrogram is passed through a vocoder, converting it back into a waveform.
  - Post-Processing Check: A check is performed to predict whether the converted voice is normal. If it passes, the final reconstructed voice is accepted; otherwise, further processing may be required.

It is important to emphasize that FEMPPDM-DVC is a speaker-dependent method. This dependency is rooted in the hypothesis that each dysarthric speaker has a unique level of speech intelligibility, along with specific patterns of articulation and phonation disruption. By tailoring the model to the individual characteristics of each speaker, FEMPPDM-DVC ensures that the converted speech closely mirrors the distinct qualities of the target dysarthric voice, leading to more accurate and personalized outcomes. Overall, the FEMPPDM-DVC method represents a sophisticated approach to voice conversion, combining advanced probabilistic modeling with detailed speaker-specific adjustments. This integration not only enhances the intelligibility of the converted speech but also preserves the individuality of each speaker, making it a powerful tool for addressing the complex needs of individuals with dysarthria.

### 2.1. Dataset and Pre-Processing

For training the proposed FEMPPDM-DVC method, we utilized a subset of the Saarland University Voice Disorder (SVD) 32 dataset, curated by the Saarland University Institute of Speech. This dataset includes over 2000 utterances from individuals with various types of dysarthria, as well as from healthy speakers, covering both male and female voices. We utilize 80-dimensional Mel-spectrograms generated through a Short-Time Fourier Transform (STFT) with a window size of 46.4 ms and a hop size of 11.6 ms. During training, the decoder processes 128 frames, while at inference, Mel-spectrograms are zero-padded to ensure the frame count is a multiple of 4. For the purpose of our analysis and visualization, we focused on 15 distinct speech types, each representing a different form of dysarthria or normal speech. These 15 categories were carefully selected to ensure a diverse representation of the speech characteristics present in the dataset. Each speech sample was resampled to 16 kHz, and the dataset was then randomly divided into 80%/10%/10% as training, testing, and validation sets, respectively.

### 2.2. Application of Fuzzy EM Clustering in Voice Conversion

To enhance the accuracy and effectiveness of the voice conversion process, we implement the Fuzzy Expectation Maximization (FEM) clustering method. This approach segments the speech data into distinct clusters, which are likely indicative of various dysarthria types. For each speech data point, the FEM algorithm calculates its membership degree with respect to both normal speech and the different dysarthric speech types. This involves analyzing the membership degree vectors assigned to each data point based on the FEM clustering results. The voice conversion process iteratively transforms the input speech until a key condition is met: the membership degree of a data point to normal speech exceeds that of any dysarthric speech type. At this stage, the transformation is considered complete, and the iteration terminates. This ensures that the output speech closely resembles normal speech patterns while effectively mitigating the effects of dysarthria. Figure 1 illustrates the structure of the FEMPPDM-DVC framework, highlighting the role of FEM clustering within the overall conversion process. Algorithm 1 provides the pseudocode for the FEM clustering stage within the FEMPPDM-DVC method.
**Algorithm 1**: FEMPPDM-DVC (Fuzzy Expectation Maximization Clustering Stage)Input:
**Data**: Speech data containing features extracted from the voice samples.**K**: Number of clusters.**MFCC Features**: Mel-frequency cepstral coefficients extracted from the speech data.**Phoneme Predictions**: Predicted phoneme features from the speech data.
Initialization:
Randomly initialize the centers for ***K*** − 1 clusters using the MFCC features.Set the center for the ***K***-th cluster as the mean of the normal speech data.
Repeat Until Convergence:
**E-step: Compute Memberships**For each data point i and each cluster j:
$$\mu_{ij}=\frac{1}{\sum_{k=1}^{K}{\left(\frac{d_{ij}}{d_{ik}}\right)}^{\frac{2}{m-1}}}$$
◆$\mathrm{where}\;d_{ij}$ is the distance between data point i and cluster center j◆m is the fuzziness coefficient◆K is the number of clusters$\mathrm{Store}\;\mathrm{the}\;\mathrm{membership}\;\mathrm{values}\;\mu_{ij}$ in the membership matrix *U*.**M-step: Update Cluster Centers**For each cluster j:
◆$\mathrm{Update}\;\mathrm{the}\;\mathrm{cluster}\;\mathrm{center}\;C_{j}$ based on the membership values of all data points, MFCC features, and phoneme predictions.◆Return the updated cluster centers.**Convergence Criteria:**Calculate the distance between the previous and current cluster centers.If the distance is less than a predefined threshold:
◆Convergence is achieved.
Otherwise:
◆Continue the iteration.

Output:
Final cluster centers.Membership matrix *U* for each data point.


This algorithm optimizes the clustering process by iteratively updating the membership values and cluster centers, ensuring that the clusters formed are well-aligned with the underlying speech data characteristics. The convergence check ensures the stability and accuracy of the clustering process, ultimately contributing to the robustness of the FEMPPDM-DVC method.

### 2.3. Inference Stage

In the inference process, the input waveform undergoes a transformation using Short-Time Fourier Transform (STFT) to produce a Mel-spectrogram, a key representation used in speech processing. The state-of-the-art Duta-VC model, which is based on a Diffusion Probabilistic Model (DPM), is then employed to perform the conversion of dysarthric speech.

The conversion process in Duta-VC involves two key phases:

1. Forward Diffusion: Noise is gradually added to the input Mel-spectrogram, simulating the progression of dysarthric speech characteristics.
2. Reverse Diffusion: This phase focuses on noise reduction, effectively reversing the forward diffusion process. The goal here is to restore the Mel-spectrogram to a form that closely resembles normal speech patterns.

Once the speech is restored through the reverse diffusion process, it undergoes clustering based on the fuzzy membership values obtained from the Fuzzy Expectation Maximization (FEM) clustering method. The membership degree of the speech being classified as “normal” is then assessed. If this degree surpasses that of other dysarthric speech types, the conversion process is considered complete, and the final output is generated.

This inference process ensures that the converted speech is not only intelligible but also as close to normal speech as possible, thereby enhancing the overall quality and effectiveness of dysarthria voice conversion.

### 2.4. Evaluation Metrics and Methodology

In order to comprehensively assess the performance of the proposed voice conversion model, a combination of subjective and objective evaluation metrics will be employed. These include the Mean Opinion Score (MOS) for subjective quality assessment, the Word Error Rate (WER) for objective performance measurement, and ablation experiments to evaluate the contribution of individual components to the overall system performance.

1. Mean Opinion Score (MOS)
  The Mean Opinion Score (MOS) is a widely used subjective evaluation metric in the fields of speech synthesis, voice conversion, and audio quality assessment. This method involves human listeners rating the quality of audio output on a predefined scale, typically ranging from 1 (unacceptable) to 5 (excellent). The MOS represents the average of all individual ratings and provides insight into how well the converted speech is perceived by users.
  In the context of voice conversion, MOS evaluations are particularly useful because they capture human perceptions of speech naturalness, intelligibility, and overall sound quality, which are difficult to measure through purely objective metrics. Multiple listeners will be recruited to evaluate the converted speech, ensuring that the MOSs reflect a broad consensus on the quality of the output.
  The MOS serves as a critical metric for gauging the subjective quality of the voice conversion model, offering insights into how well the synthesized speech mimics human-like speech characteristics.
2. Word Error Rate (WER)
  The Word Error Rate (WER) is an objective evaluation metric that measures the accuracy of the system’s speech conversion by comparing the generated text with a reference or ground truth transcription. WER is a common metric in tasks, such as speech recognition, machine translation, and text correction, and it quantifies how well the system transcribes or converts speech compared to a reference standard.
  WER is calculated based on three key operations:
  - Substitution (S): Replacing a correct word with an incorrect one.
  - Deletion (D): Omission of a word that should have been present.
  - Insertion (I): Addition of an extraneous word that does not belong.
  The formula to compute WER is:
  $$WER=\frac{S+D+I}{N}\times 100\%$$
  where N is the total number of words in the reference transcription. A lower WER implies a more accurate voice conversion, indicating fewer mistakes in the output. It provides an objective measure to assess how closely the system’s output matches the expected result and serves as a benchmark to compare the performance of various models.
3. Ablation Experiments
  The ablation experiment is a technique used to evaluate the contribution of different components or features in a model by systematically removing or altering them and observing the impact on overall performance. In the context of voice conversion, this experiment is designed to assess the importance of individual modules or processing steps, such as feature extraction, diffusion models, or clustering algorithms, in the conversion process.
  By selectively disabling or modifying specific components of the voice conversion pipeline, we can understand how each part contributes to overall performance in terms of both subjective (MOS) and objective (WER) metrics. The ablation study helps identify which parts of the system are most critical to achieving high-quality speech conversion, thereby guiding further improvements and optimizations.

The combination of MOS, WER, and the ablation experiment ensures a thorough evaluation of the proposed voice conversion system from multiple perspectives. The MOS offers a subjective assessment based on human judgment, WER provides an objective measure of transcription accuracy, and the ablation experiment highlights the importance of individual model components. Together, these metrics form a comprehensive framework for evaluating the system’s performance and guiding future enhancements.

## 3. Result and Discussion

**Figure 2:**
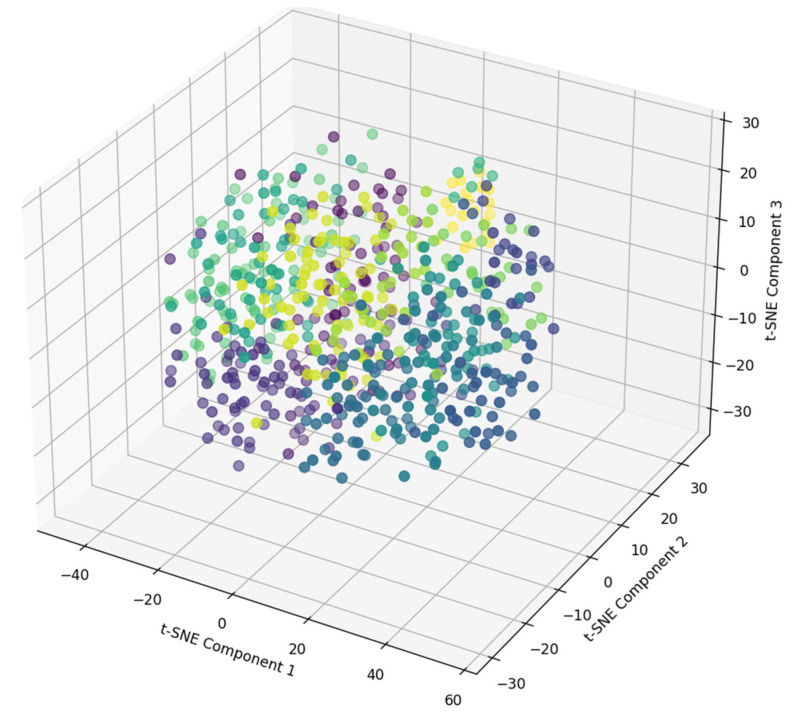
Three-dimensional t-SNE visualization of speech type distributions.

Figure 2 displays a three-dimensional t-SNE representation of 15 different speech types. t-SNE is employed here to reduce the dimensionality of the feature space, which allows for a more interpretable visualization of the relationships and similarities between the various speech types.

The 3D t-SNE plot provides a comprehensive view of how 15 different speech types are distributed across three principal components. Each point in the scatter plot represents an individual speech sample, with colors indicating distinct clusters or speech types. The spread and separation of clusters in the visualization reflect the degree of similarity or dissimilarity among the speech types, where tightly packed clusters suggest a higher within-type similarity, and more separated clusters indicate distinct differences between speech types.

This visualization highlights the method’s ability to maintain clear distinctions between various types of dysarthric and normal speech after applying the FEMPPDM-DVC model. It also serves as evidence for the model’s clustering efficacy, supporting the overall goal of achieving more accurate and personalized voice conversion.

**Figure 3:**
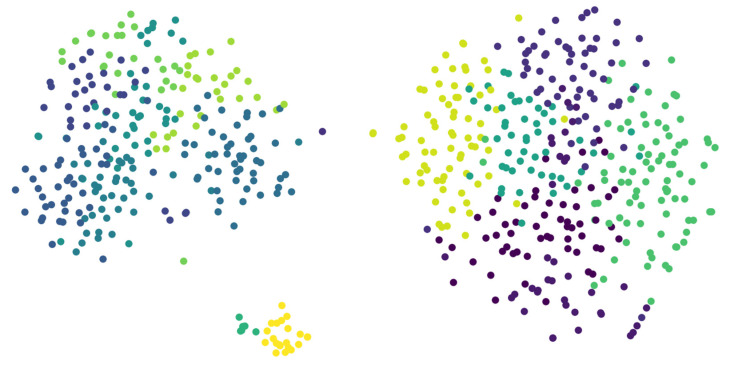
Two-dimensional t-SNE visualization of MFCC-based speech clustering.

Figure 3 provides a 2D t-SNE plot which visualizes the distribution of speech data after clustering based on MFCC (Mel-frequency cepstral coefficients) features. The clustering process initially involved extracting 256-dimensional features from the speech data, which were then subjected to traditional hard clustering techniques. The high-dimensional clusters were subsequently reduced to two dimensions using t-SNE (t-Distributed Stochastic Neighbor Embedding) for better visualization and interpretation. In this plot, each point represents a speech sample, and the colors indicate different clusters that were identified during the hard clustering process. The distinct groupings in the 2D space suggest that the MFCC features effectively captured the variability in the speech data, allowing for a clear separation between different clusters. The two large, well-separated clusters on the right and left sides of the plot likely correspond to significantly different speech types, while the smaller cluster near the bottom center indicates a speech type that is less prevalent or more distinct from the others.

The t-SNE visualization reveals that the MFCC features were effective in distinguishing between different speech types, as evidenced by the formation of coherent clusters. This visualization not only validates the use of MFCC features for speech classification but also demonstrates the effectiveness of the clustering approach in identifying distinct speech types. The clear separation of clusters in the 2D space supports the hypothesis that the proposed method can accurately group speech samples based on their underlying characteristics, providing a solid foundation for further analysis or processing tasks, such as voice conversion or dysarthria classification.

Juvenile dysphonia manifests in various articulation errors, including sound substitutions (e.g., saying “wabbit” instead of “rabbit”), omissions (e.g., saying “at” or “ca” instead of “cat”), distortions (e.g., saying “thun” instead of “sun”), and additions (e.g., saying “buh-lue” instead of “blue”). These speech anomalies are often due to issues with the coordination of tongue and mouth muscles, improper auditory feedback, or rhythm and prosody disorders that affect the natural flow of speech.

The two visualizations provided illustrate the acoustic characteristics of speech affected by juvenile dysphonia, a condition that affects the proper articulation of certain sounds during speech.

**Figure 4:**
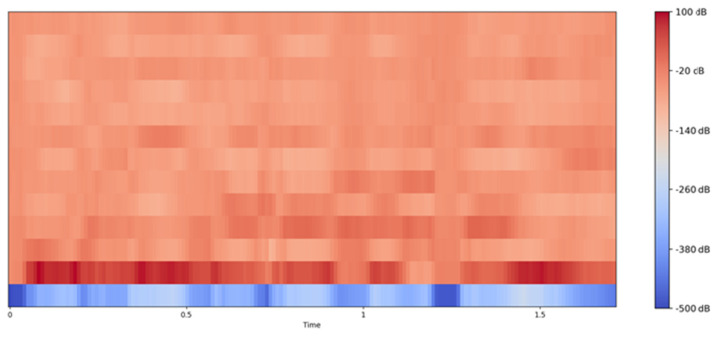
MFCC visualization of juvenile dysphonia.

- MFCC Visualization:

Figure 4 displays the Mel-Frequency Cepstral Coefficients (MFCC) of a speech sample from a person with juvenile dysphonia. MFCCs are commonly used in speech recognition tasks to capture the timbral aspects of the sound. In this visualization, the MFCCs are plotted over time, with the color intensity representing the amplitude of the coefficients. The plot indicates variations in the speech signal’s spectral properties over time, highlighting specific areas where the speech deviates from typical patterns, which is common in cases of speech disorders.

**Figure 5:**
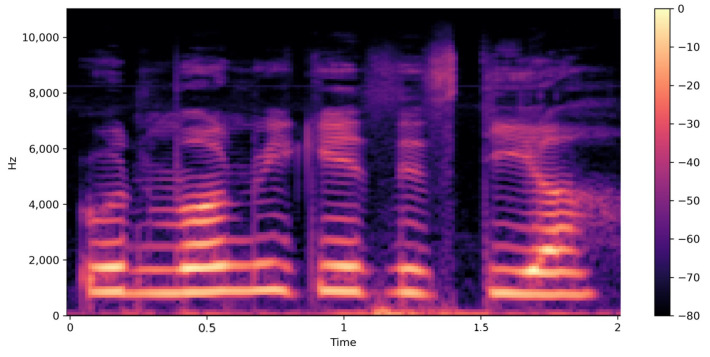
Mel-spectrogram visualization of juvenile dysphonia.

- Mel-spectrogram Visualization:

Figure 5 is a Mel-spectrogram of the same speech sample. This plot visualizes the frequency content of the speech signal over time, focusing on the Mel scale, which is designed to mimic the human ear’s perception of sound. The Mel-spectrogram provides a detailed representation of the speech signal’s harmonic structure and temporal evolution. In the context of juvenile dysphonia, this visualization helps to identify anomalies in sound production, such as distorted or missing frequencies, which correspond to the articulation errors described in the condition.

The MFCC and Mel-spectrogram visualizations together offer a comprehensive view of how these articulation errors manifest in the acoustic domain. The MFCC plot reveals the spectral energy distribution over time, while the Mel-spectrogram provides a more detailed frequency-based analysis. Both tools are essential for diagnosing and understanding the severity and nature of speech disorders like juvenile dysphonia, and they play a crucial role in the development of corrective speech therapy techniques.

- Results of MOS Evaluation

The following table presents the results for naturalness, intelligibility, and speaker similarity based on the MOSs. These scores were averaged across the 25 selected samples from the SVD dataset and compared with other existing voice conversion methods, such as StarGAN-VC [44], Auto-VC [45], CycleGAN-VC [46], VAE [47,48], CycleVAE [49], and Duta-VC [16]. The performance of the proposed method is highlighted. The results are shown in Table 1.

1. Naturalness: The proposed method achieves a score of 3.44, slightly outperforming Duta-VC (3.42) and significantly outperforming all other methods, indicating that the proposed model produces speech that is more natural and less robotic.
2. Intelligibility: With a score of 3.54, the proposed method also surpasses other models in terms of improving speech clarity, making it more intelligible than models like Auto-VC and CycleGAN-VC. This improvement suggests that the proposed voice conversion method is highly effective in preserving or enhancing the clarity of the converted speech.
3. Speaker Similarity: The proposed method achieves the highest score of 3.96, outperforming Duta-VC (3.94) and all other compared models. This indicates that the converted speech is very similar to the target speaker’s voice, supporting the success of the arbitrary-to-arbitrary conversion goal.

These results demonstrate that the proposed method excels in all three evaluation categories, particularly in maintaining high levels of naturalness, intelligibility, and speaker similarity, surpassing other contemporary voice conversion techniques. The superior performance in these areas confirms the effectiveness of the proposed approach in generating high-quality, speaker-consistent converted speech.

- Word Error Rate (WER) Comparison for Mild and Severe Dysarthria

WER is a critical objective metric in speech processing, used to measure the accuracy of speech recognition systems or voice conversion models by comparing the system’s output with a reference transcription. In this evaluation, WER measures how effectively each model can reconstruct intelligible speech from dysarthric speech, with a lower WER indicating better performance. The Automated Speech Recognition (ASR) system was employed in the pre-trained espnet toolkit5. This CTC-attention hybrid encoder-decoder network is used to perform the WER evaluation. The WER result is shown in Table 2.

For mild cases, the proposed method achieved the lowest WER of 0.507, followed closely by Duta-VC with 0.527. In contrast, other models like Auto-VC and CycleGAN-VC exhibited higher WER scores, with Auto-VC reaching 0.78, indicating that these models struggle more in generating intelligible speech for mild dysarthria cases.

For severe dysarthria, the WER of the proposed method remained competitive, recording a score of 0.548, again outperforming the Duta-VC model, which had a WER of 0.554. The other GAN-based models, such as CycleGAN-VC (0.83) and StarGAN-VC (0.751), exhibited noticeably higher error rates, particularly for severe cases.

In the total WER comparison, which averages results across both mild and severe cases, the proposed method achieved the best overall performance, with a WER of 0.5275, further demonstrating its robustness across different levels of dysarthria severity. Other models, such as Auto-VC (0.8235) and CycleGAN-VC (0.8055), showed consistently higher error rates, suggesting that GAN-based models still have limitations when applied to dysarthria voice conversion tasks.

The superior performance of the proposed method in both mild and severe dysarthria cases can be attributed to the diffusion-based approach utilized in this study. This approach likely offers greater stability and precision in converting the unique speech features of dysarthric individuals, such as vocal fry, breathiness, and articulation difficulties. In contrast, the limitations of GAN-based models for dysarthria voice conversion may stem from their difficulty in handling the complex and distorted speech patterns that characterize dysarthric speech.

By analyzing the WER results, it is evident that the diffusion-based method outperforms traditional GAN-based techniques, particularly for severe dysarthric cases where speech distortion is more pronounced. This suggests that diffusion-based voice conversion offers a more accurate and reliable solution for restoring intelligibility in dysarthric speech, as reflected in its lower WER scores.

**Figure 6:**
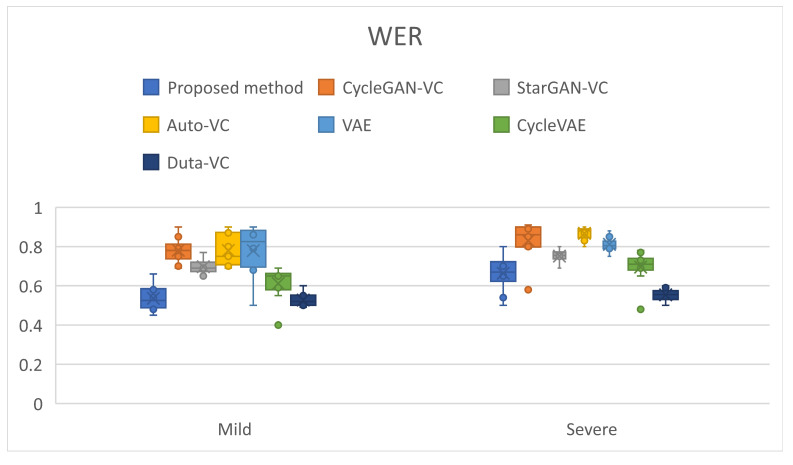
Word Error Rate (WER) comparison across different voice conversion models for mild and severe dysarthria.

Figure 6 showcases the WER scores for different voice conversion models across mild and severe dysarthria cases. Lower WER scores indicate better performance in producing intelligible speech from dysarthric inputs. The proposed diffusion-based method consistently outperforms other models across both mild and severe dysarthria cases, highlighting its efficacy in handling speech impairment-related challenges.

- Ablation Experiments

**Figure 7:**
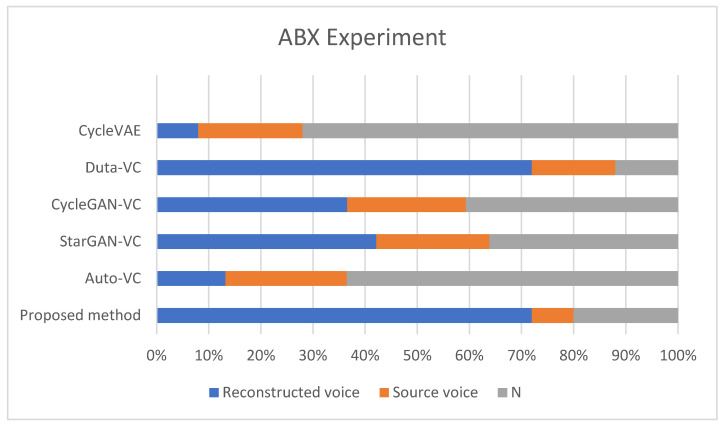
Effect of removing speaker-encoder on naturalness and intelligibility.

The ablation experiments aimed to determine how much the speaker-encoder contributes to maintaining speech quality. By eliminating the speaker-encoder, the model’s ability to preserve naturalness (how closely the output resembles natural human speech) and intelligibility (how understandable the speech is) was evaluated. Figure 7 illustrates the listener voting results for each model.

Model-Specific Observations:

1. CycleVAE: Exhibited the least effective performance, with around 10% of votes favoring the reconstructed voice. This poor result is likely due to the lack of feature extraction relevant to naturalness, causing significant degradation in both naturalness and intelligibility.
2. Duta-VC: Demonstrated moderate performance, with about 50% of votes favoring the reconstructed voice. The absence of the speaker-encoder moderately affects the speech quality, though the model still manages to retain some degree of intelligibility.
3. CycleGAN-VC: Achieved roughly 40% preference for the reconstructed voice, suggesting that the model relies heavily on the speaker-encoder to maintain voice characteristics.
4. StarGAN-VC: Showed similar results to CycleGAN-VC, with approximately 45% of votes favoring the reconstructed voice. The absence of the speaker-encoder results in a noticeable degradation in naturalness.
5. Auto-VC: The absence of the speaker-encoder severely impacted Auto-VC’s performance, with about 60% of votes leaning towards the source voice. This indicates that the speaker-encoder plays a vital role in retaining the naturalness of the converted voice.
6. Proposed Method (diffusion-based): Achieved the best results, with around 70% of votes in favor of the reconstructed voice. Even without the speaker-encoder, the proposed method retains superior performance in both naturalness and intelligibility, indicating its robustness. The diffusion-based approach effectively manages speech conversion, even without the direct input of speaker features, which highlights its strength in complex voice conversion tasks.

The results of this ablation experiment reveal the critical role that the speaker-encoder plays in most GAN-based models. The absence of this module leads to a significant drop in naturalness and intelligibility, as indicated by the higher preference for the source voice in models like Auto-VC, CycleGAN-VC, and StarGAN-VC. However, the proposed diffusion-based method continues to perform well, suggesting that it can handle intricate voice conversion tasks with greater robustness.

## 4. Conclusions

In this paper, we explored the application of a novel method called Fuzzy Expectation-Maximization Phoneme Prediction in Diffusion Model-based Dysarthria Voice Conversion (FEMPPDM-DVC). Our proposed approach specifically targets the challenges associated with dysarthria voice conversion by seamlessly integrating Mel-frequency cepstral coefficients (MFCC) features and a phoneme predictor. This integration allows for a more personalized and adaptable voice conversion process, tailored to meet the unique needs of individuals with articulation disorders.

FEMPPDM-DVC represents a significant advancement in improving communication quality for individuals who struggle with articulation issues. By leveraging the strengths of both MFCC features and phoneme prediction, our method provides a more accurate and user-specific conversion of dysarthric speech, potentially enhancing the day-to-day communication experience for those affected by such disorders.

In this study, we have proposed and evaluated a novel voice conversion approach designed to improve both naturalness and intelligibility, specifically focusing on dysarthric speech. The proposed method, based on diffusion models, demonstrates superior performance compared to traditional GAN-based approaches. Our evaluation, through both Mean Opinion Score (MOS) and Word Error Rate (WER) metrics, shows that the diffusion-based model excels in delivering more natural and intelligible voice conversion, especially for severe dysarthria cases where GAN-based models tend to struggle.

The results from the ablation experiments further highlight the robustness of the diffusion-based method. Even without the inclusion of a speaker-encoder, the model maintains its effectiveness in preserving speaker characteristics and producing intelligible outputs. This contrasts sharply with GAN-based models like CycleGAN-VC and StarGAN-VC, which experience a significant drop in performance when the speaker-encoder is removed. The ABX test results reinforce this conclusion, as the proposed method consistently outperformed other models in terms of listener preference for the reconstructed voice.

Future work will aim to refine the diffusion-based approach by further optimizing model parameters for real-time voice conversion applications. We also plan to expand the dataset to include a wider range of dysarthria severity levels and speaker variations to further validate the generalizability of our model. Additionally, the integration of complementary techniques, such as automatic speech recognition (ASR) and fine-grained feature extraction, will be explored to enhance both the precision and adaptability of the proposed voice conversion framework.

In conclusion, the proposed diffusion-based voice conversion method sets a new benchmark in the field of dysarthria speech rehabilitation. Its resilience in the absence of a speaker-encoder, coupled with its strong performance across subjective and objective metrics, makes it a promising tool for real-world applications where naturalness, intelligibility, and speaker similarity are paramount. The findings of this study lay the groundwork for further exploration into diffusion models and their potential to transform voice conversion and rehabilitation technologies.

## Figures and Tables

**Table 1:** The MOS comparison of reconstructed speech.

	Naturalness	Intelligibility	Speaker Similarity
Source voice	3.00	3.46	3.84
StarGAN-VC	1.71	2.08	2.23
Auto-VC	1.64	1.94	2.08
CycleGAN-VC	1.55	1.65	2.00
VAE	1.86	2.98	1.92
CycleVAE	2.06	2.70	2.82
Duta-VC	3.42	3.33	3.94
Proposed method	3.44	3.54	3.96

**Table 2:** The WER comparison of other method.

	Mild	Severe	Total
StarGAN-VC	0.697	0.751	0.724
Auto-VC	0.78	0.867	0.8235
CycleGAN-VC	0.781	0.83	0.8055
VAE	0.779	0.867	0.823
CycleVAE	0.612	0.695	0.6535
Duta-VC	0.527	0.554	0.5405
Proposed Method	0.507	0.548	0.5275

## Data Availability

The datasets used and/or analyzed during the current study available from the corresponding author on reasonable request.
